# Micropulse Laser Therapy as an Integral Part of Eye Disease Management

**DOI:** 10.3390/medicina59081388

**Published:** 2023-07-28

**Authors:** Flaviu Bodea, Simona Gabriela Bungau, Mihaela Alexandra Bogdan, Cosmin Mihai Vesa, Ada Radu, Alexandra Georgiana Tarce, Anamaria Lavinia Purza, Delia Mirela Tit, Cristian Bustea, Andrei-Flavius Radu

**Affiliations:** 1 Doctoral School of Biomedical Sciences, University of Oradea, 410087 Oradea, Romania; dr.flaviu.bodea@gmail.com (F.B.); v_cosmin_15@yahoo.com (C.M.V.); dtit@uoradea.ro (D.M.T.); andreiflavius.radu@uoradea.ro (A.-F.R.); 2 Department of Pharmacy, Faculty of Medicine and Pharmacy, University of Oradea, 410028 Oradea, Romania; purza_lavinia@yahoo.com; 3 Department of Preclinical Disciplines, Faculty of Medicine and Pharmacy, University of Oradea, 410073 Oradea, Romania; 4 Ducfarm Pharmacy, 410514 Oradea, Romania; adaroman96@gmail.com; 5 Medicine Program of Study, Faculty of Medicine and Pharmacy, University of Oradea, 410073 Oradea, Romania; tarce_alexandra@yahoo.com; 6 Department of Surgery, Oradea County Emergency Clinical Hospital, 410169 Oradea, Romania; cristibustea@yahoo.com

**Keywords:** micropulse laser therapy, ocular diseases, diabetic macular edema, glaucoma, laser treatment, ophthalmology

## Abstract

Ocular diseases can significantly impact vision and quality of life through pathophysiological alterations to the structure of the eye. The management of these conditions often involves a combination of pharmaceutical interventions, surgical procedures, and laser therapy. Laser technology has revolutionized many medical fields, including ophthalmology, offering precise and targeted treatment options that solve some of the unmet needs of other therapeutic strategies. Conventional laser techniques, while effective, can generate excessive thermal energy, leading to collateral tissue damage and potential side effects. Compared to conventional laser techniques, micropulse laser therapy delivers laser energy in a pulsed manner, minimizing collateral damage while effectively treating target tissues. The present paper highlights the advantages of micropulse laser therapy over conventional laser treatments, presents the implications of applying these strategies to some of the most prevalent ocular diseases, and highlights several types and mechanisms of micropulse lasers. Although micropulse laser therapy shows great potential in the management of ocular diseases, further research is needed to optimize treatment protocols, evaluate long-term efficacy, and explore its role in combination therapies.

## 1. Introduction

Ocular diseases comprise a broad spectrum of conditions that can have severe impacts on vision and quality of life. Diabetic macular edema (DME), retinal vein occlusion (RVO), central serous chorioretinopathy (CSCR), age-related macular degeneration (AMD), primary open-angle glaucoma (POAG) [1,2], and secondary glaucoma, such as pseudo exfoliative glaucoma (PXG) [3], constitute some of the most prevalent ocular disorders. These conditions present significant management challenges and necessitate the development of novel therapeutic strategies to adequately preserve the condition of or treat ocular diseases.

The management of ocular diseases may involve a combination of pharmaceutical interventions, surgical procedures, and laser therapy. Anti-vascular endothelial growth factor (anti-VEGF) drugs, corticosteroids, and intraocular pressure-lowering medications have played a significant role in controlling inflammation, reducing macular edema, and regulating intraocular pressure in these conditions [4]. In more advanced cases, surgical interventions such as vitrectomy and trabeculectomy have also been utilized [5].

In the past few decades, the treatment framework for ocular diseases has undergone remarkable advancements, and laser therapy is one approach that has shown considerable potential. Laser technology has revolutionized the field of ophthalmology by providing precise and targeted treatment options [6,7]. In particular, micropulse laser therapy (MPLT) has emerged as a novel approach with enormous potential for the treatment of ocular diseases [8].

Laser therapy has been an integral element of the management of ocular diseases for decades. For retinal photocoagulation, panretinal photocoagulation, and trabeculoplasty, various laser approaches, including argon, krypton, and diode lasers, have been utilized with varying degrees of success. However, conventional laser techniques frequently generate excessive thermal energy, resulting in collateral tissue injury and potential side effects. Different retinal conditions have been treated for many years with conventional laser photocoagulation. The inner retina and retinal pigment epithelium (RPE) can be thermally damaged, resulting in a noticeable whitening of the retina. Besides contributing to the desired therapeutic outcome, the procedure may also cause undesirable side effects such as choroidal neovascularization (CNV) in the vicinity of the laser scar, epiretinal fibrosis, and visual field abnormalities [6,9].

Considering a perceptible burn on the retina caused by continuous-wave radiation, the traditional laser approach has a number of drawbacks, including reduced visual acuity, enlarging scars, and subretinal fibrosis [10]. In contrast, MPLT constitutes an important breakthrough in laser technology. The subthreshold diode micropulse laser (SDM) was developed to increase the effectiveness and decrease these negative effects. This method delivers the energy by splitting the beam into several brief pulses (i.e., 100–300 µs), offering two advantages: a shorter exposure duration along with a subvisible clinical outcome. Every pulse has an “on and off” time frame (i.e., duty cycle (DC)), allowing the tissues to cool before the following pulse [11].

The specified amount of laser energy is provided in the conventional continuous-wave manner by a single 0.1–0.5 s laser pulse. For the micropulse mode, a series of repeated brief laser pulses distributes laser energy inside an “envelope” with a standard width of 0.1 to 0.5 s. Every pulse lasts approximately 100–300 µs. The “envelope” consists of both “off” time, which is the interval between the micropulses, and “on” time, which is the length of every micropulse. The “off” duration is crucial because it allows the previously emitted heat to cool. The time interval, *T*, is equal to the total of the “on” and “off” timings, and 1/*T* represents the frequency (i.e., pulses per second), *f,* expressed in hertz (Hz). The ratio of “on” time to period *T* is referred to as the DC in percentages [12].

An important step for the overall management of ocular diseases was the development of a micropulse mode of action for retinal lasers, applied in subthreshold power settings [11]. In fact, the micropulse mode is a characteristic of the lasers that are available on the market with several wavelengths, including 532 nm, 577 nm, and 810 nm. The laser is applied in numerous, brief, repeated impulses expressed in microseconds, with breaks that allow the cooling of the retinal tissue. In retinal impairments, the efficient time frame in which a laser functions is empirically calibrated to 5% with a 0.2 s exposure envelope length [8].

Subthreshold therapy using lasers aims to avoid traces on the retina (i.e., spots that might be seen using any diagnostic equipment at hand), including optical coherence tomography (OCT), fundus autofluorescence, fundus angiography, and biomicroscopy. According to medical investigations, there is no evidence of photoreceptor or RPE impairment following subthreshold micropulse laser therapy (SML) [13].

When treating retinal or macular illnesses, micropulse laser treatment (MPLT) is an alternative to the traditional continuous-wave laser. The therapeutic result generated with the subthreshold micropulse laser is not followed by thermal retinal damage, unlike the conventional laser. This aspect is especially crucial when a procedure close to the fovea is necessary. The most prevalent indications for micropulse therapy include DME, CSCR, and macular edema caused by RVO [12].

MicroPulse technology decreases temperature buildup in nearby non-target tissues by breaking a continuous laser beam of energy into smaller “on” pulses and prolonged “off” pauses, reducing the injury to surrounding tissues. Many different primary and secondary glaucomas have been treated with transscleral cyclophotocoagulation (TSCPC) administered using the MicroPulse treatment technique, including angle-closure glaucoma (ACG), congenital iridocorneal endothelial syndrome, and normo-tensive, post-keratoplasty, aphakic, pseudoexfoliative, steroid-induced, POAG, uveitic, neovascular, and post-vitrectomy glaucoma [14].

The present narrative review article seeks to provide a comprehensive overview of MPLT as a revolutionary approach for the treatment of ocular diseases, with an emphasis on its distinctive characteristics and contributions to the field in comparison with conventional laser strategies. In addition to providing an in-depth evaluation of the current state of knowledge, this paper is aimed at contributing to the scientific understanding of MPLT by exploring the clinical implementation via data from medical studies and discussing the underlying mechanisms and current utilization of the different types of subthreshold laser treatment.

## 2. Research Methodology

**Figure 1:**
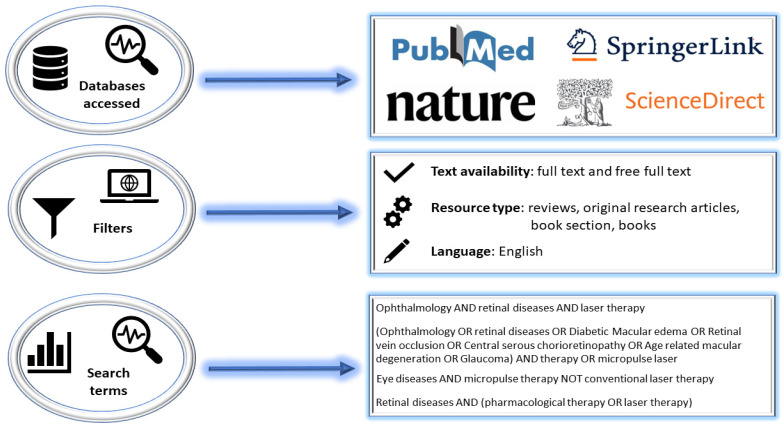
Methodology for searching for and filtering bibliographic resources.

The present paper evaluates the scientific literature that has addressed MPLT interventions in ocular pathologies (i.e., DME, RVO, CSCR, AMD, POAG, and PXG), with emphasis on their management, clinical trial data, and mechanisms and parameters of function, along with a focus on improving the state of knowledge in the field by updating, organizing, and centralizing new information in this area. In this regard, databases with wide coverage and validation in terms of the content of publications, including in the medical field (i.e., PubMed [15], Nature [16], SpringerLink [17], and ScienceDirect [18]), were accessed and consulted. The advanced search methodology (Figure 1) required the use of Boolean logical operators (i.e., AND, OR, and NOT) and certain search filters to filter the numerous data displayed by these broad databases and to include as valid bibliographic resources only eligible manuscripts with the required search and paper design conditions. Before screening, papers that were not written in English, were not very informative, or were not article-type, books, or book sections were eliminated. A total of 112 bibliographic references from 1979 to 2023 were chosen and referenced to validate the information provided in this paper.

## 3. Types and Mechanisms of Micropulse Laser Strategies

With an established sequence of on and off intervals, subthreshold micropulse lasers aim to supply energy at a predetermined section of the exposure period. DC, which represents the laser’s actual delivery time, is described as the ratio of on time to exposure time (on + off). Individual spots are fundamentally unnoticeable when using ophthalmoscopy as well as any currently available multimodal imaging techniques for diagnosing the retina (i.e., OCT angiography, spectral domain-OCT, retromod imaging, and fundus autofluorescence), and they never cause a local decrease in retinal sensitivity when assessed with microperimetry [19].

The DC of the laser is reduced in the micropulse laser in order to attain this subthreshold application. The laser energy is split up into multiple brief repeated pulses instead of a single continuous pulse, typically lasting between 100 and 300 µs with 1700–1900 µs between each pulse. As a result, the laser’s DC is essentially reduced to 5–10% of the value provided by a typical laser. The retinal tissue is able to dissipate the built-up heat to prevent the threshold of apoptosis and cell death through prolonged rest intervals between each laser micropulse. Using optical and electron microscopy, it has been shown that micropulse power as low as 10% of the threshold power can cause localized alterations in the RPE without harming the neurosensory retina [20].

It is possible to reduce the absorption of energy and thermal diffusion towards the neurosensory retina by using a near-infrared diode laser with a longer wavelength of 810 nm. The RPE, which has become increasingly recognized as the source of powerful extracellular variables that are operating as disease mediators, could be targeted using greater laser energies and photothermal effects with less thermal retinal damage by micropulsing the 810 nm diode laser and decreasing the frequency of laser micropulses by lengthening the “off-time” between micropulses throughout the exposure envelope [21].

The early attempts to decrease laser intensity using continuous-wave krypton, argon, and diode lasers are referred to as “classical” subthreshold photocoagulation. The retinal burns were referred to as “subthreshold” or even “invisible” since they were significantly less noticeable when compared to white, full-thickness retinal burns in accordance with ETDRS criteria. The lesions of “classical” subthreshold photocoagulation were consistently noticeable both clinically and through fundus fluorescein angiography at the moment of treatment and thereafter. In fact, these lesions were “threshold” (i.e., photocoagulation damage limited to the outer retina and therefore not as obvious at the moment of treatment) or perhaps less severe suprathreshold (i.e., full-thickness retinal photocoagulation typically effortlessly discernible at the moment of treatment) [22,23].

Glaucoma can be treated with a non-invasive laser procedure referred to as micropulse transscleral laser therapy. The MicroPulse P3 Delivery Device (Iridex, Mountain View, CA, USA) and Cyclo G6 Laser (Iridex, Mountain View, CA, USA) reduce tissue temperature increase and coagulative injury by using an infrared diode laser with a wavelength of 810 nm that divides the continuous energy wave into a series of pulses. According to the DC, the laser is “on” for 31.3% of the procedure and “off” for the remaining time (68.7%). In comparison to the conventional TSCPC technique, the MicroPulse technology produces a lower cumulative energy application and, when paired with a sweeping technique, gives a more homogenous energy distribution. Micropulse transscleral laser therapy reduces the risk of problems as opposed to continuous-wave TSCPC through lower temperature targets and improved thermal management [24].

The 1990s saw the development of subthreshold diode micropulse lasers, which used the rapid application of a burst of laser pulses with pulse durations of 100–300 microseconds throughout a 100–500 ms time frame. Recent advances have led to the development of SRT, a method that uses a burst of 1.4 ms laser pulses with a 10 ms gap in between each pulse. Despite the fact that the laser pulses in both subthreshold diode micropulse and SRT applications raise the RPE’s temperature (i.e., have thermal effects), there is a reduced risk of heat diffusion into neighboring tissues, such as the neural retina, because there is enough time between each pulse for the temperature to potentially return to baseline. It is estimated that the RPE has a thermal relaxation interval of around 10 ms, which is a measurement of how well thermal energy may flow through the cell. This means that a small amount, if any, of thermal energy migration into photoreceptors would occur when there are gaps between pulses longer than 10 ms [25].

In order to send 3 nanosecond (ns) pulses to the posterior eye, a laser in the nanosecond range called 2RT^®^, designed by Ellex Pty Ltd. in Adelaide, Australia, uses a Q-switched frequency-doubled laser [26].

The concept of the micropulse laser served as inspiration for the creation of the nanosecond laser, which has a pulse energy of 0.2% of that of a regular laser. The DC in this instance is significantly shorter. According to Brinkmann et al., the mechanism causing nanosecond laser-induced RPE cell injury is the production of momentary microbubbles surrounding melanosomes following the intracellular plasma boiling temperature, which is comparable to the pulse length of a micropulse laser [27].

**Figure 2:**
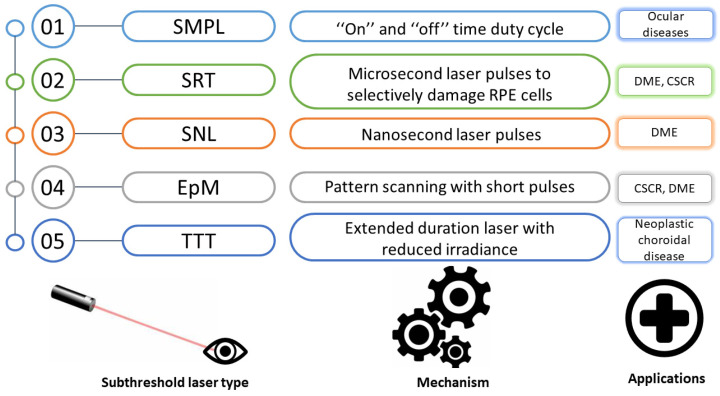
Principles and current applications of the various subthreshold laser treatment types. SMPL, subthreshold micropulse laser; SRT, selective retina therapy; SNL, subthreshold nanosecond laser; EpM, endpoint management; TTT, transpupillary thermotherapy; CSCR, central serous chorioretinopathy; DME, diabetic macular edema; RPE, retinal pigment epithelium.

Figure 2 presents the mechanisms and current medical applications of the different sorts of subthreshold laser treatment [28].

## 4. Diabetic Macular Edema

The focus of contemporary treatments for DME is on reducing the impact of VEGF, even though focal/grid macular laser treatment was historically regarded as the gold standard treatment for DME. Recent studies have shown that intravitreal injections of anti-VEGF drugs, regardless of whether they are monotherapy or combined with focal/grid macular laser treatment, are more effective than focal/grid laser treatment alone for the treatment of DME [29].

The prior gold standard of conventional treatment for clinically significant (CS) DME was standard retinal laser photocoagulation. While diffuse macular edemas are treated using a grid pattern, focal treatments are used for treating regions with focal edema and leakage [30].

The Early Treatment Diabetic Retinopathy Study (ETDRS) found that CS DMEs that underwent focal argon photocoagulation with a perceptible burn after a three-year observation experienced a reduction in moderate vision loss by 50% [31].

In order to treat center-involved DME (CI-DME), it is important to determine the safety and effectiveness of using a micropulse macular laser in conjunction with intravitreal aflibercept. A prospective, randomized, controlled, single-blind study was conducted to address this issue; it included 30 eyes from 30 individuals with CI-DME with best-corrected visual acuity (BCVA) ranging from 20/30 to 20/400 being randomly assigned into two groups. Intravitreal aflibercept injections (IVT-AFL) were administered to patients in the first group along with a fake laser. The second group received a micropulse laser and IVT-AFL. For 48 weeks, both groups were monitored every 4 weeks, and retreatment was carried out based on a pro re nata approach in agreement with the predefined standards. This approach is a treatment strategy in which patients receive additional injections or interventions depending on their individual requirements or therapeutic response. The average number of intravitreal injections administered to each group at 48 weeks served as the primary outcome indicator. Variations in central macular thickness (CMT) and BCVA at 24 and 48 weeks were considered secondary result indicators. At 48 weeks, both groups received, on average, a similar number of intravitreal injections. The BCVA and CMT of both groups also showed improvement at this time interval. Nevertheless, there was no statistically significant difference in the levels of amelioration between the groups [32].

Table 1 shows a series of medical data comparing MPLT with conventional laser strategies obtained in different types of studies following the evaluation of patients with DME. Moreover, most study results show the superior efficacy of MPLT compared to strategies using conventional lasers.

A recently published trial assessed the outcomes of combining MPL and anti-VEGF therapy, showing great potential [38]. Using this treatment combination, Khattab and colleagues demonstrated that it might be both efficient and secure. Following the treatment, the frequency of aflibercept injections reduced while the structural and visual effects remained the same [39].

According to research by Abouhussein and colleagues, the adjuvant use of a 577 nm micropulse laser with aflibercept reduced the number of injections while still being successful in treating DME that had not yet received treatment. Reduced annual injection frequency is possible by using the treatment combination as opposed to intravitreal injections alone [40].

A recent systematic study on the use of intravitreal injections and subthreshold MPL to treat macular edema was published by Gawecki, showing that subthreshold diode micropulse laser plus anti-VEGF therapy may require fewer intravitreal injections than anti-VEGF monotherapy with equivalent functional and morphological outcomes [41].

Following a review of numerous studies on MPLT and anti-VEGF in DME, it was concluded that using MPLT in combination with anti-VEGF would result in non-inferior functional and morphological results compared to those of anti-VEGF monotherapy in cases of limited macular edema while reducing the number of intravitreal injections needed. It was indicated that more extensive randomized studies were required to define the precise function of MPLT in the management of DME [42].

Table 2 presents relevant medical data from different studies targeting the efficacy profile of MPLT in DME treatment.

## 5. Retinal Vein Occlusion

The effectiveness of subthreshold micropulse diode laser photocoagulation (SMDLP) for DME and/or macular edema secondary to branch retinal vein occlusion (BRVO) has been the subject of numerous investigations [11].

Thirty-two subjects (thirty-two eyes) with macular edema secondary to BRVO underwent treatment with SMDLP to evaluate its effectiveness for persistent macular edema secondary to BRVO, comprising BCVA > 20/40. Prior to receiving treatment, all patients had been monitored for at least 6 months after the initial diagnosis of their condition. The subjects were divided into groups 1 (BCVA ≤ 20/40) and 2 (BCVA > 20/40) based on their initial Snellen visual acuity. Stable BCVA at 6 months and a decrease in CMT on OCT were the primary result indicators. At 6 months, BCVA in the entire participant sample did not change considerably, whereas CMT did. At 3 months, CMT showed little or no modification in group 1, but at 6 and 12 months, CMT showed considerable decreases. Group 2 showed a significant decrease in CMT at 6 months after starting treatment and a very slight decrease at 3 months. SMDLP appears to reduce macular edema with little retinal damage in individuals with persistent macular edema related to BRVO. According to recent research, BRVO subjects with BCVA greater than 20/40 may benefit from SMDLP treatment for macular edema [52]. Furthermore, SMDLP has been suggested as a potential treatment for DME in an effort to reduce the negative effects of standard grid laser photocoagulation [33].

Ideally, because SML stimulates RPE by producing cytokines and increasing pumping activity, it should decrease inflammation and enhance fluid removal. Compared to other retinal vascular disorders, RVO may exhibit more severe inflammatory conditions and vascular hyperpermeability. This is possibly why the results of SML in macular edema secondary to RVO are not always satisfactory. In these circumstances, it is unlikely that SML will be more effective than intravitreal steroids or anti-VEGF therapy [8].

A study that comprised 153 patients who were treated at Shijiazhuang People’s Hospital and were given a diagnosis of non-ischemic central RVO (CRVO)-induced ME was conducted between January 2019 and January 2021. On this premise, the experimental group received laser treatment, whereas the control group received only conbercept treatment. The CMT was assessed through OCT prior to and at one and three months following treatment, and the BCVA was calculated using the internationally accepted logarithm of the minimum angle of resolution (logMAR) chart. During the three-month follow-up (FU) period, complications, including conjunctival bleeding and increased intraocular pressure (IOP), if present, were documented. Patients with non-ischemic CRVO may benefit from conbercept in terms of their BCVA and CMT, and when used in association with MPLT, these improvements are greater than they were prior to treatment. Additionally, there were statistically significant variations between the experimental group and the control group. There were no significant adverse responses reported in either group, and the prevalence of the complications was comparable [53].

According to the findings of Bougatsou et al., MPLT is effective in treating CS macular edema that is not centrally involved. Similar to earlier studies’ findings, these results also showed that conbercept paired with MPLT was more successful in enhancing BCVA and CMT for individuals with non-ischemic CRVO [34].

In another clinical study, two subgroups of patients were treated with intravitreal ranibizumab (i.e., 27 subjects) and subthreshold micropulse laser (i.e., 24 patients). In the intravitreal ranibizumab group, the average number of treatments was 3.81, while in the subthreshold micropulse laser group, it was 1.5. In terms of the BCVA mean score at the starting point, at 1, 6, and 12 months, the subgroups were comparable. At 1, 6, and 12 months, there was a statistically significant difference between the baseline values, subthreshold micropulse laser, and intravitreal ranibizumab groups for the mean CMT. According to the findings of the study, there was no difference between intravitreal ranibizumab and yellow subthreshold MPLT for macular edema caused by BRVO in terms of reducing macular thickness and improving VA over the course of a year. These findings suggest that a feasible alternate strategy for treating macular edema caused by BRVO could involve the use of a subthreshold micropulse laser [54].

Table 3 presents data from clinical trials evaluating SMPLT treatment of macular edema secondary to retinal vein occlusion.

## 6. Central Serous Chorioretinopathy

Focal laser photocoagulation does not appear to have any discernible effects on the improvement in visual acuity or the recurrence rate during follow-up [59].

The two procedures that are most frequently used to treat CSCR are subthreshold MPLT and photodynamic therapy (PDT) with verteporfin. However, PDT may raise the risk of subsequent CNV, choroidal ischemia, and RPE atrophy [60]. The application of PDT in the treatment of acute CSCR may be restricted because of these side effects. Recent studies have shown that half-dose PDT together with verteporfin is an efficient treatment for acute CSCR, decreasing subretinal fluid and improving VA in most subjects while reducing side effects. However, it is expensive and not available everywhere [61].

Subthreshold micropulse as a laser emission method uses trains of repeatedly delivered ultrashort laser pulses. It produces a sublethal cellular thermal action at the RPE without transferring heat to the nearby retinal tissue [62].

A total of thirty-nine patients with acute CSCR were included in the study. A subthreshold micropulse laser was used to treat 18 individuals, while half-dose PDT was used to treat 21 patients. BCVA according to the ETDRS diagram, the percentage of eyes with completely cleared subretinal fluid, the quantity of treatments, and retinal sensitivity through microperimetry over the course of the 12-month period under investigation were the primary outcome measures. Throughout the 12-month interval, the subthreshold micropulse laser group had an average of 1.6 treatments, compared to 1.3 for the half-dose PDT group. At 12 months, the subthreshold micropulse laser group had an 83.3% complete resolution rate compared to a 90.5% complete resolution rate in the half-dose PDT group. The average BCVA was 76.62 ± 11.57 for the half-dose PDT group and 75.28 ± 12.58 for the subthreshold micropulse laser group at 12 months following treatment. Throughout the 12-month follow-up, there was no statistically significant variation between the two groups’ average retinal sensitivity values [63].

Atrophic alterations with paracentral scotoma, CNV development, and widening of the laser scar throughout the course of the FU are all possible side effects of conventional laser treatment for CSCR. Because of these latter side effects, conventional laser treatment can be used only on extramacular and extrafoveal leaky spots that are at least 500 microns away from the fovea. The use of traditional laser applications in routine clinical practice has decreased significantly with the development of PDT subthreshold laser therapy [64].

Although focal laser photocoagulation expedites subretinal fluid resolution for both acute and chronic CSCR, data regarding the final visual result and recurrence rate have varied between investigations [65].

Ficker et al. found similar recurrence rates, ultimate visual acuity, and color discrimination in their study of 44 eyes with FU ranging from 6.4 years to 12.1 years [66]. The results of micropulse laser (810 nm) and argon laser (514 nm) treatments were compared at 12 weeks in a randomized controlled study conducted by Verma et al., which randomized 30 patients with CSCR that presented a single focal leak. Both groups showed complete fluid resolution and comparable end BCVA, though the contrast sensitivity for the diode laser group was significantly higher than that for the argon laser group, where it improved from a baseline value of 130.66 to a final value of 215.33 as opposed to a mean absolute contrast sensitivity enhancement of 98.4 to a final average value of 306.0 in the diode group. Additionally, the diode group had no persistent scotomas, while the argon laser group showed 20% incidents of persistent scotomas [67].

The objective of one prospective investigation was to estimate the efficacy of SML in CSCR patients by considering several morphological factors. Moreover, 31 patients were evaluated after the disease onset, and SML was carried out on patients with persistent subretinal fluid at 3 months. In CSCR patients, a favorable response to SML may be associated with a reduced persistent subretinal fluid, a narrower pigment epithelial detachment, and a reduction in the number of hyperreflective foci [68].

In CSCR patients, an open-label, multicenter, randomized controlled clinical trial compared the morphological and functional safety and effectiveness of half-dose photodynamic therapy (89 patients) versus high-density SML treatment (90 patients). Half-dose photodynamic therapy was demonstrated to be more beneficial than high-density SML in the treatment of CSCR, resulting in a substantially greater number of patients with resolution of subretinal fluid and improvements in functionality [69].

When 26 eyes were treated with a diode laser in a different series, Chen et al. observed that complete fluid resolution occurred in 14 out of 15 eyes with focal leaks, but only in 5 out of 11 eyes experiencing diffuse leakage [62].

In another study, the main objective was to assess the effectiveness of CSCR trans- foveal subthreshold MPLT administered at least six months after the onset of the condition. Moreover, 32 patients with CSCR with durations ranging from 3 weeks to 6 months were included in the study. Following each subthreshold MPLT session, all patients received trans-foveal subthreshold MPLT and were monitored for a minimum of 3 months. If the initial round of therapy did not produce the desired results, a total of two subthreshold MPLT sessions were scheduled. BCVA and retinal morphology were the evaluation criteria. In 26 cases (81.25%), subretinal fluid completely disappeared. Following therapy, the final BCVA considerably increased. A better final BCVA was generally associated with early subthreshold MPLT. BCVA increased in eyes that achieved a complete resolution of subretinal fluid [70].

The possibility of treating CSCR with subthreshold MPLT using a 532 nm (532-SML) wavelength has been proposed in a retrospective study. Prior to and 12 weeks after treatment, spectral-domain OCT and BCVA parameters, including the sub-foveal outer nuclear layer, choroidal thickness, CMT, ellipsoid band, interdigitation band, subretinal fluid, and external limiting membrane, were assessed. A total of 26 eyes were included. Neither the spectral domain OCT measurements nor visual acuity showed any appreciable changes. Nevertheless, 42.3% (*n* = 11) of the subjects experienced visual benefits, and subretinal fluid was totally reabsorbed in 50% of the cases. Complications were not observed [71].

A total of 34 eyes from 34 individuals with acute CSCR who either received 577 nm SML therapy (SML group, *n* = 16 eyes) or were simply observed (observation group, *n* = 18 eyes) were included in a comparative retrospective case series. A 6-month timeframe was used to collect the data, and the sub-foveal choroidal thickness, CMT, and BCVA were all observed. At 1 month, 3 months, and 6 months, the SML group demonstrated a BCVA improvement that was considerably larger than that of the observation group. Moreover, the SML group had a considerably larger CMT reduction at 1 month, 3 months, and 6 months [72].

Table 4 presents data on the efficacy of SMPLT in the management of CSCR, demonstrated by significant results in major clinical trials.

## 7. Micropulse Laser Therapy for Age-Related Macular Degeneration

The evolution of age-related geographic atrophy (ARGA) was examined using a pan-macular low-intensity/high-density subthreshold diode micropulse laser. To establish the speed of radial linear ARGA progression, both during observation and after the pan-macular subthreshold diode micropulse laser treatment, the retinal images of every eye with ARGA in the formerly mentioned records, which include all eyes active in a vitreoretinal practice electronic medical record that present dry AMD, were selected and examined. Furthermore, 67 eyes of 49 ARGA patients were monitored both before and after the beginning of SDM therapy. The radius of ARGA lesions developed by 1 to 540 µm per year before treatment and 44 to 303 µm per year after regular pan-macular subthreshold diode micropulse laser commencement, according to the masked measurement for treatment versus monitoring. As a result, after pan-macular SDM, the rate of radial linear advancement dropped by 47% annually. No unfavorable therapeutic results were observed [86].

There is currently no highly effective treatment for the regression of AMD-related reticular pseudo drusen (RPD). A prospective investigation aimed to assess the safety and short-term efficacy of SML in patients with RPD secondary to dry AMD. A total of 20 eyes from 20 patients were examined in one study, and it was concluded that SML appears to be a safe treatment for RPD caused by dry AMD based on short-term safety results and may be able to induce RPD regression [87].

In a retrospective study, 21 eyes from 16 patients with intermediate AMD and drusenoid pigment epithelial detachment (D-PED) were sequentially evaluated. The purpose of this investigation was to assess the long-term structural and visual outcomes of D-PED in intermediate AMD eyes treated with yellow SML at 577 nm. In contrast to the gradual development of D-PED reported by prior research, this study’s preliminary findings indicate that SML can mitigate visual loss and the risk of advancement to more severe AMD in eyes with D-PED in intermediate AMD [88].

In a referral-only retina practice, 19 consecutive eyes with severe AMD that were treated for retinal angiomatous proliferation lesions to lessen subretinal exudation were retrospectively examined. High-intensity, brief laser pulses were used to close the RCA. The visual acuity, persistence of RCA, and resolution of subretinal fluid were all regarded as outcome indicators. The average baseline visual acuity was discovered to be 20/140 and hand motion was 20/50. At the time of the initial therapy, there was subretinal exudation in every eye and 73% of them had subretinal fibrosis. The average number of laser treatment sessions for patients was 3.52 (1–12) over an average monitoring period of 11.7 (2–23) months. The final visual acuity measured was 20/146 (count fingers to 20/40) on average. The subretinal fluid completely disappeared in 53 % of the eyes. There was subretinal fibrosis in every single case. The RCA was completely closed in 43% of the cases [89].

Table 5 shows data from research examining the effectiveness of laser treatment for AMD.

## 8. Glaucoma

### 8.1. Primary Open Angle Glaucoma

TSCPC in MicroPulse mode, also known as MicroPulse Transscleral Laser Therapy (MPTLT), is a non-incisional laser therapy utilized for treating various forms of glaucoma. The MicroPulse P3 Delivery Device, also known as the MicroPulse P3 Probe (Iridex, Mountain View, CA, USA), is used in conjunction with the Cyclo G6 Laser (Iridex, Mountain View, CA, USA) to deliver an 810 nm infrared diode laser in a transscleral manner. According to the theory behind MPTLT, it offers a better safety profile than conventional continuous-wave transscleral laser cyclophotocoagulation, besides significantly decreasing IOP, enabling its use in a wider range of individuals [91].

Seventy-two Chinese subjects with POAG underwent micropulse diode laser trabeculoplasty (MDLT) with a 532 nm laser system to perform a 360° angle treatment, as described recently by Hong et al. A total of 19 patients with initial POAG from this group were not taking any medication. The average IOP was 20.6 mmHg before MDLT and 16.5 mmHg on average after MDLT, then it stayed stable over the following 24 weeks. Additionally, no IOP spikes were noted, and the average number of glaucoma medications per patient decreased dramatically from 1.7 to 1.5, suggesting that MDLT is an effective and safe method for treating POAG [92].

The first argon laser trabeculoplasty (ALT) procedure was developed by Wise and Witter in 1979 [93]. According to the results of the glaucoma laser experiment, ALT is just as effective as topical beta blocker medication, if not more efficient [94]. Selective laser trabeculoplasty (SLT), which provides an IOP decrease comparable to ALT with greater precision treatment and less tissue loss, was first presented in 1995 by Latina et al. Subsequently, research has shown that SLT is just as successful as monotherapy with drops [95,96].

In order to observe the decreasing IOP in patients with open-angle glaucoma, one study compared the tolerability, safety, and effectiveness of micropulse laser trabeculoplasty MDLT with SLT. In total, 31 patients received 360° SLT and 38 individuals received 360° MDLT as part of the overall randomization. Patients with ACG, uveitic glaucoma, neovascular glaucoma, or end-stage glaucoma were not included. An IOP drop of at least 20.0% or more than 3 mmHg from the starting point was considered a treatment reaction. At 24–52 weeks, IOP decreased to 3 mmHg from the initial value in 37.0% of the micropulse group and 36.0% of the selective laser cohort of participants. Moreover, during the 24–52-week period, the IOP decreased by 20.0% from baseline in 36.0% of selective laser subjects and 29.6% of micropulse subjects. At every time point up to 52 weeks after therapy, both groups showed comparable drops in IOP as percentage and absolute values declined from baseline. The micropulse group noted more treatment failures up to 52 weeks following the procedure, although this difference was not statistically relevant. Both during and following the surgery, the micropulse group experienced less pain [97].

There are few long-term studies contrasting the classic SLT with the more recent version, which uses a laser emitting at 532 nm. The following is a comparison of MPLT with SLT made by scientists to evaluate their efficacy and safety. MPLT was administered to 43 consecutive eyes, and SLT was administered to a total of 85 consecutive eyes. Subjects with open-angle glaucoma who were undergoing their first laser trabeculoplasty procedure were enrolled in the study. The MPLT group’s initial IOP was 18.0 mmHg with an average of 1.8 glaucoma medicines, while the SLT group’s initial IOP was 18.2 mmHg with a mean of 2.0 medicines. The SLT group experienced larger transitory IOP rises an hour after receiving the laser treatment. At one year, there was a likelihood that the SLT group would report greater results than the micropulse laser therapy group [98].

In a retrospective investigation of eighty-four eyes, the researchers used substantially greater fluence values than the previously published frequently used settings (i.e., up to 400 J/cm^2^, or eight times the average fluence presented in the literature) and demonstrated a significant decrease in IOP and medication use. In the study, 41% of the participants exhibited varying degrees of vision loss. However, it is important to note that the FU period of the study was relatively short, which might not have provided a comprehensive understanding of the temporary nature of some of the observed vision loss during the investigated period. The majority of patients had severe glaucoma, and almost 70% had already undergone incisional glaucoma surgery [99].

The use of micropulse transscleral cyclophotocoagulation (MP-TSCPC) in patients with excellent vision was reported for the first time in the following investigation. At every postsurgical FU visit, it was revealed that MP-TSCPC considerably decreased glaucoma medication use and IOP, and neither of these factors significantly affected visual acuity. A retrospective analysis was performed for subjects who received MP-TSCPC at the Ross Eye Institute and the Mayo Clinic between July 2016 and August 2017 and had a minimum of three months of monitoring and had BCVA ≥20/60. MP-TSCPC was performed on 61 eyes in 46 individuals. At each subsequent examination point, the average IOP and the average number of glaucoma medicines were considerably reduced in comparison to the starting point. At 12 months, the mean IOP had decreased by 40.2% from baseline, with an IOP decrease of at least 20% in 85.4% of participants. The average amount of glaucoma medications had decreased by 0.82 ± 0.53, with an IOP decrease in 79.6% of participants [100].

### 8.2. Secondary Glaucoma: Pseudoexfoliative Glaucoma

In several nations, such as Ireland, Norway, Oman, and Saudi Arabia, PXG accounts for more than half of open-angle glaucoma incidences. It is distinct from POAG in pathophysiology, clinical manifestations, future outcomes, and therapeutic management. It usually manifests asymmetrically and is linked with a higher peak and mean IOP, increased diurnal IOP fluctuations, and a reduced IOP tolerance [101].

Modern laser techniques, such as micropulse laser trabeculoplasty, require thorough evaluation in PXG patients, but they indicate effectiveness in reducing IOP by approximately 20% from baseline in around half of PXG eyes up to 1 year after laser therapy [102].

POAG and PXG patients treated with anti-glaucoma drugs that demanded supplemental IOP reduction were included in a prospective, single-center study. Moreover, 532 nm micropulse laser trabeculoplasty was used to treat the eyes in a 360-degree treatment.

The investigation included 20 eyes from 20 POAG patients and 18 eyes from 18 PXG patients. Both POAG and PXG eyes treated with micropulse laser trabeculoplasty evidenced statistically significant reductions in IOP relative to baseline. Several parameters like the endothelial cell count, central corneal thickness, and hexagonal cell ratio did not change significantly between baseline and 6 months after the laser intervention [103].

In one medical study evaluating the efficacy of micropulse transscleral cyclophotocoagulation, 96 patients were included. Of the total number of cases, 32 POAG, 30 PXG, and 34 cases of other categories of secondary glaucoma were identified. Micropulse transscleral cyclophotocoagulation has been demonstrated to be a similarly effective method of reducing IOP in POAG, PXG, and other varieties of secondary glaucoma patients. However, refractory secondary glaucoma patients had an elevated rate of reoperation [104].

Another study assessed the long-term effects of glaucoma treatment using MPLT at a 577 nm yellow wavelength. The medical data of 51 patients (51 eyes) who underwent 180° MPLT for the first time and had unmanaged PXG or POAG were examined. IOP reduction of at least 20% and post-treatment IOP of at least 21 mmHg were considered successful outcomes of MPLT. The case was regarded as unsuccessful if the number of drugs needed to treat the condition increased or if glaucoma surgery or additional laser trabeculoplasty were needed following therapy. Throughout monitoring, the IOP decreased by 16.72 ± 11.87% at 3 months, 15.07± 13.76% at 6 months, 12.63± 14.29% at 12 months, 16.66 ± 19.32% at 24 months, and 16.75 ± 19.78% at 36–48 months when compared to the initial evaluation [105].

Table 6 provides a comprehensive overview of studies assessing the effects of laser therapy in glaucoma.

## 9. Conclusions

MPLT has emerged as a significant and promising approach to addressing a variety of ocular diseases. This technique offers several benefits over conventional laser strategies, addressing the limitations and adverse effects of continuous-wave lasers. MPLT applies subthreshold power settings and delivers laser energy in the manner of brief pulses with intermittent breaks, resulting in less thermal energy and less collateral tissue injury. Moreover, this type of therapy protects the structural integrity of the retina. Therefore, it may represent a valuable therapeutic option for conditions such as DME, RVO, CSCR, AMD, POAG, and PXG.

Future directions should focus on expanding its application to other ocular conditions, personalizing treatment approaches, and leveraging technological advancements. MPLT represents a transformative option in ocular disease management with improved precision, safety, and treatment outcomes. Continued research and exploration of this innovative approach will lead to enhanced patient outcomes and advancements in ophthalmology.

## Figures and Tables

**Table 1:** Studies evaluating the impact of laser therapy in DME.

Type of Study	Number of Treated Eyes/ Follow-up Period (Months)	Evaluation	Results	Refs.
Prospective, randomized, double-masked	123/12	Comparison between the effectiveness of different laser treatments for DME, specifically comparing two types of laser treatments (810 nm ND-MPLT and 810 nm HD-MPLT) with a traditional photocoagulation method (mETDRS)	HD-MPLT and mETDRS photocoagulation are similarly effective in terms of BCVA and CRT outcomes for patients with CS-DME, while ND-MPLT demonstrated stable BCVA without significant retreatment needs	[33]
Prospective and randomized	60/6	Comparison of the effectiveness of two different laser treatments for DME, specifically comparing 532 nm MPLT with 532 nm focal photocoagulation	The reduction in CRT was significantly better in the MPLT group compared to the focal photocoagulation group	[34]
Prospective and randomized	116/6	Comparison of the effectiveness of two different laser treatments for DME, specifically comparing short-pulse subthreshold 532 nm laser treatment with micropulse 810 nm laser treatment	Visual acuity significantly improved in the micropulse 810 nm laser treatment group	[35]
Prospective, randomized, double-masked	266/24	Comparison of the effectiveness of two different laser treatments for CI-DME. The study compared 577 nm MPLT with the traditional modified mETDRS photocoagulation	No significant differences between the MPLT and mETDRS groups in terms of BCVA and CRT	[36,37]

DME, diabetic macular edema; ND-MPLT, nondamaging micropulse laser treatment; HD-MPLT, high-density micropulse laser treatment; mETDRS, modified early treatment diabetic retinopathy study; BCVA, best-corrected visual acuity; CRT, central retinal thickness; CS-DME, clinically significant diabetic macular edema; Cl-DME, center-involving diabetic macular edema; SMPL, subthreshold micropulse laser; NRT, nondamaging retinal laser therapy.

**Table 2:** Evaluations of the efficacy of MPLT in DME.

Type of Study	Number of Treated Eyes/ Follow-up Period (Months)	Evaluation	Results	Refs.
Retrospective	134/16	Retrospective assessment of 134 eyes with previously untreated center-involving mild DME treated with 577 nm SMPL	A decrease in the CRT and average thickness of the nine ETDRS sectors was found in comparison to baseline; no side effects were observed	[43]
	70/2	Retrospective assessment of the correlation between the severity of DME and the efficacy of subthreshold yellow NRT	NRT provided an improvement in BCVA and CRT	[44]
	56/3	Treatment with 577 nm MPLT	Significant improvement in BCVA	[45]
	80/6		Significant decrease in CRT and stabilization of BCVA	[46]
	9/3		Significant decrease in CRT and stabilization of BCVA	[47]
Prospective	52/12		Significant improvement in BCVA and ETDRS and a significant decrease in hyper-reflective retinal spots and microaneurysms	[48,49]
	75/6		Significant reduction in CRT and a stabilization of BCVA	[50]
	20/6		Significant improvement in BCVA and an important reduction in CRT	[51]

DME, diabetic macular edema; SMPL, subthreshold micropulse laser therapy; MPLT, micropulse laser therapy; EDTRS, early treatment diabetic retinopathy study; BCVA, best-corrected visual acuity; CRT, central retinal thickness; NRT, nondamaging retinal laser therapy.

**Table 3:** Effects of SMPLT treatment on macular edema secondary to branch retinal vein occlusion.

Type of Study	Number of Treated Eyes/ Follow-up Period (Months)	Evaluation	Results	Ref.
Prospective randomized	35: 18- SGLT group; 17-IVB group/12	Comparison of the effectiveness of SMPLT vs. IVB in recurrent ME secondary to BRVO	The IVB group showed a significant reduction in CRT, from 484 µm to 271 µm, as well as an improvement in BCVA; no significant effects were observed in the SMPLT group	[55]
	36: 17 SMPLT group; 19 LPC group/24	Comparison of the effectiveness of different laser treatments for macular edema secondary to BRVO occurring three to eighteen months prior	At the 12-month follow-up, there were no significant differences between the groups in terms of BCVA, CRT, or MV; at the 24-month follow-up, 65% of patients who underwent SMPLT treatment and 58% of patients who received LPC treatment showed an improvement of at least 10 letters in visual acuity	[56]
	24: 13 SMPLT group; 1 SMPLT + IVT group/12	Comparison of the effectiveness of SMPLT and SMPLT + IVT	After 12 months, the combined treatment of SMPLT and IVT resulted in a significant improvement of at least 10 letters in visual acuity for 91% of patients, whereas the SMPLT-alone group showed a 62% improvement with a statistically significant difference between the two groups	[57]
Retrospective, consecutive, case–control study	46: 22 IVR + SMPLT group 24—IVR group/6	Comparison of the effectiveness of IVR + SMPLT vs. IVR in eyes with ME secondary to BRVO, treatment-naïve; assessment of CRT, BCVA	Both groups showed a significant improvement in BCVA and CME, with no significant difference between the two groups; the number of injections in the group receiving IVR was statistically higher (2.3) compared to the group receiving IVR plus SMPLT	[58]
Retrospective	51: 27 IVR group 24 SMPLT group/12	Comparison of the effectiveness of SMPLT vs. IVR in patients with ME secondary to BRVO, BCVA, CRT; the frequency of treatments was assessed after a minimum of 3 months from the occlusive event	There were no significant differences in the final BCVA or CRT between the two groups; the IVR group received an average of 3.81 treatments, while the SMPLT group received 1.5 treatments	[54]
Retrospective, interventional case series	32: BCVA ≤ 20/40 group BCVA > 20/40 group/12	Comparison of the effectiveness of SMPLT in eyes with longstanding ME after BRVO (at least 6 months)	Both groups showed a significant decrease in CRT, with no notable difference between them; there was no significant improvement in BCVA in either group	[52]

BRVO, branch retinal vein occlusion; BCVA, best-corrected visual acuity; CME, cystoid macular edema; CRT, central retinal thickness; IVB, intravitreal bevacizumab; IVR, intravitreal ranibizumab; IVT, intravitreal triamcinolone; LPC, laser photocoagulation; ME, macular edema; MV, macular volume; SMPLT, subthreshold micropulse laser treatment; SGLT, subthreshold grid laser treatment.

**Table 4:** Findings from key clinical trials evaluating the efficacy of SMPLT in managing CSCR.

Study	No of Eyes/Duration of CSCR (Months)	Evaluation	Outcomes	Ref.
Prospective, Interventional, noncomparative	26/>4	Provide information on the visual and clinical effects in chronic idiopathic CSCR with juxta foveal leaking of subthreshold diode micropulse laser photocoagulation	BCVA improved, in a statistically relevant manner, in 100% of patients, increasing in 57.7% of cases by at least three lines and in 23.1% of cases by one to three lines	[62]
	24/>3	For treating CSCR, it is important to confirm the effectiveness of nonvisible micropulse diode laser irradiation	BCVA enhancement	[73]
	15/≥3	Provide information on the safety and effectiveness of micropulse 577 nm yellow laser treatment in the therapeutic management of persistent conditions of CSCR	A 100% decrease in SRF; after three months and six months, respectively, total SRF absorption was 73% and 86%; BCVA increased from 0.67 Snellen to 0.85 Snellen	[74]
	10/≥3	Analyze the effectiveness of guided yellow microsecond laser in the treatment of persistent CSCR sub-foveal leaks	SRF decrease was 100%; total SRF resorption at six months was 60%, CRT decreased from a mean of 298 to 215 µm, and the variation in BCVA from 73.3 to 76.9 letters ETDRS at six months was not statistically significant	[75]
	39/>3	The anatomic result, visual outcomes, and the safety profile of the treatment for chronic CSCR and the evaluation of the long-term effectiveness of 577 nm sub-threshold micropulse yellow laser treatment; 17.82 months average follow-up (13–24 months)	A decrease in the mean CRT from 369 μm to 250 μm; an enhancement in BCVA in 89.7% of cases	[76]
Prospective, comparative, controlled	52: 16 SDM group; 10 BCZ group; 26 observation group/>3	Comparison of the effectiveness in CSCR of SDM vs. BCZ	When treating CSCR, SDM photocoagulation was more effective than intravitreal injections with 1.25 mg BCZ, which determined an improvement in macular perimetry and visual acuity	[77]
Prospective, randomized, double-blind, sham-controlled pilot trial	15: 5 sham group; 10 SDM group/≥6	In individuals with chronic CSCR, evaluation of 810 nm SDM laser	When compared to the sham group at 3 months, BCVA was considerably improved in the intervention group	[78]
Retrospective	11/≥113	The macular thickness and visual results in individuals with symptomatic chronic CSCR modification after therapy with an 810 nm subthreshold micropulse diode laser	Following SMLT, the maximum macular thickness reduced by values between 20 μm and 338 μm; the median BCVA boost was six letters ETDRS	[79]
	15/>3	Analyze the safety and effectiveness of one session of chronic CSCR therapy using a subthreshold micropulse yellow laser (577 nm)	A 100% decrease in SRF, 40 percent in SRF overall resorption, anda statistically significant enhancement in BCVA from 20/40 Snellen to 20/30 Snellen	[80]
	10/≥6	Evaluate the effectiveness of subthreshold micropulse yellow laser photocoagulation on the short term for treating chronic CSCR	BCVA enhanced from 0.21 logMar to 0.035 logMar, while CRT decreased from 349.2 m to 261.2 m	[81]
	38/>1.5	For evaluating the effects of treatment for individuals with chronic CSCR, a 577 nm SMLT was used	A 74% decrease in SRF; CRT was typically reduced by 115 μm, while BCVA was improved by 0.06 logMAR	[82]
	11/1–7 (3.6 on average)	Evaluate the outcomes of the CSCR therapy with low-intensity/high-density SDM	Improvement in BCVA from an average 20/37 to an average of 20/24, with a CRT decrease from mean 508 µm to average 250 µm	[83]
	51/>4	Analyze the functional and morphological consequences, as well as the variables affecting the visual outcome, in individuals with chronic CSCR who have had SMLT	SRF was completely absorbed in 70.6%; CRT was reduced on average from 337.6 to 260 µm; a statistically relevant mean BCVA +0.08 logMAR improvement	[84]
	29: 15 conventional laser group; 14 SML group />3	Comparatively assess the effectiveness of conventional lasers vs. SML in treating focal retinal pigment epithelium leakages in individuals suffering from CSCR	SML had therapeutic results comparable to conventional lasers, although retinal pigment epithelium damage was avoided in eyes treated with CSCR. Total SRF resorption was reported in 64.3% of the SML group; CRT decreased from 328 μm to 192 μm, on average. Statistically negligible BCVA fluctuation from 0.96 to 0.94 Snellen	[85]

BCVA, best-corrected visual acuity; BCZ, intravitreal bevacizumab; CSCR, central serous chorioretinopathy; CRT, central retinal thickness; ETDRS, early treatment diabetic retinopathy study; SDM, subthreshold diode micropulse; SMLT, subthreshold micropulse laser therapy; SRF, subretinal fluid.

**Table 5:** Scientific investigations assessing the effects of laser therapy in AMD.

Type of Study	Number of Treated Eyes/ Follow-up Period (Months)	Evaluation	Results	Ref.
Retrospective	13/3–7 (average 5)	Evaluating the efficacy of subthreshold diode micropulse laser therapy in eyes with a lack of response to all anti-vascular endothelial growth factor drugs, encompassing a history of at least three consecutive ineffective aflibercept injections	Out of the total eyes studied, 92% demonstrated improvement, with 69% (9 out of 13) achieving complete resolution of macular exudation; visual acuity remained unaffected, while notable improvements were observed in both central and maximum macular thicknesses	[90]
Prospective non-randomized	20/3	Assessment of the safety and short-term effectiveness of subthreshold laser treatment in patients with reticular pseudo drusen secondary to dry age-related macular degeneration	During the follow-up, there was a noteworthy rise in the number of Stage 1 reticular pseudo drusen, accompanied by a substantial decrease in Stage 3 reticular pseudo drusen; analysis of the outer nuclear layer thickness revealed a significant increase after the treatment, which was linked to reticular pseudo drusen regression; the findings suggest that subthreshold laser treatment as an end-point management approach appears to be safe for reticular pseudo drusen secondary to dry age-related macular degeneration, with positive short-term safety outcomes	[87]

**Table 6:** Studies evaluating the impact of laser therapy in glaucoma.

Type of Study	Number of treated Eyes/ Follow-up period (Months)	Evaluation	Results	Ref.
Prospective interventional case series	30/6	Assessing the effectiveness of laser treatments for primary open-angle glaucoma, with 77 nm laser equipment with defined therapy parameters	Baseline IOP was 18.07 ± 1.91 mmHg, on average; the IOP dropped rapidly and significantly to 14.17 ± 1.56 mmHg at six months after baseline	[106]
Prospective, comparative, randomized	31: 16 MDLT group; 15 ALT group/3	Comparison of the effectiveness of two different laser treatments for evaluating the IOP decreasing benefits and safety in open-angle glaucoma subjects treated with ALT and MDLT (810 nm)	At three months, ALT dramatically reduced IOP, while MDLT did not; diode laser trabeculoplasty dramatically reduced the proportion of eyes having an IOP decrease of 20% or more compared to argon laser trabeculoplasty; the inflammation of the anterior segment caused by MDLT was not severe, and its safety profile appeared to be favorable	[107]
Prospective	20/12	Assessment of individuals with OAG that was medically unsupervised, the pressure-lowering advantages of subthreshold MDLT	In 75% of OAG eyes with inadequate medical management, MDLT successfully decreased IOP without any major side effects	[108]
	48/6	Assessing the effectiveness of laser treatments with one session with unilateral MLT therapy that used a 577 nm diode laser to treat the trabecular meshwork across 360 degrees for lowering medication burden or IOP	At six months after the laser treatment, MLT had a low failure rate, negligible post-laser inflammation, and was successful in lowering IOP and prescription drugs in OAG	[109]
	69: 38 MLT group; 31 SLT group/3	Examining the effectiveness, acceptability, and safety of SLT against MLT in lowering IOP in unmanaged open-angle glaucoma	With less pain experienced both before and after the surgery, micropulse trabeculoplasty showed similar effectiveness to SLT in a 52-week monitoring interval	[110]
Retrospective	40/12	Analyzing the results of 180-degree MDLT in glaucoma patients who have secondary open-angle glaucoma	In individuals with open-angle glaucoma, 180° MDLT is a safe but unsuccessful procedure	[111]
	30/5.33	Assessing the effectiveness of the 810 diode Optos FastPulse laser (34 cycles of treatment) for primary open-angle glaucoma in patients with medical greatest intervention failing; it was evaluated how the pressure changed one hour after the therapy	IOP decreased by 17.2% from the starting point pre-laser at a mean post-laser reduction of 3.2 mmHg; over varied time intervals, ranging from several weeks to a few months, the IOP dropped steadily	[112]

ALT, argon laser trabeculoplasty; MDLT, micropulse diode laser trabeculoplasty; MLT, micropulse laser trabeculoplasty; OAG, open-angle glaucoma; SLT, selective laser trabeculoplasty.

## Data Availability

Data are contained within the article.
